# Efficacy evaluation of Wenxin Keli in treating anthracycline-induced atrial arrhythmias (WARMA study): protocol for a multicenter, randomized, double-blind, placebo-controlled clinical trial

**DOI:** 10.3389/fcvm.2026.1805789

**Published:** 2026-05-18

**Authors:** Linke Jiao, Na An, Xinye Li, Xiaoling Liu, Lei Miao, Ge Hu, Yuchen Jiang, Xiaochen Yang, Yonghong Gao, Hongcai Shang, Yanwei Xing

**Affiliations:** 1 Department of Cardiology, Guang’anmen Hospital, China Academy of Chinese Medical Sciences, Beijing, China; 2 Key Laboratory of Chinese Internal Medicine of Ministry of Education, Dongzhimen Hospital, Beijing University of Chinese Medicine, Beijing, China; 3 Institute of Acupuncture and Moxibustion, China Academy of Chinese Medical Sciences Beijing, China; 4 Department of Cardiology, Qilu Hospital of Shandong University, Jinan, China; 5 Department of Cardiology, Jinqiu Hospital of Liaoning Province, Shenyang, China; 6 Department of Lymphoma, Tianjin Medical University Cancer Institute and Hospital, Tianjin, China; 7 Dongfang Hospital, Beijing University of Chinese Medicine, Beijing, China

**Keywords:** anthracycline, atrial arrhythmias, cardiotoxicity, randomized controlled trial, Wenxin Keli

## Abstract

**Background:**

Anthracycline-induced cardiotoxicity is a major factor affecting the long-term prognosis of cancer patients. Atrial arrhythmias, as common manifestations of cardiotoxicity, significantly impair quality of life and may compromise the effectiveness of anticancer therapy, representing a significant clinical challenge. Wenxin Keli (WXKL), a traditional Chinese medicine formulation with the effects of replenishing qi, nourishing yin, and promoting blood circulation, has demonstrated potential anti-arrhythmic benefits. However, high-quality evidence supporting its application in the field of cardio-oncology remains limited. This study aims to systematically evaluate the efficacy and safety of WXKL in patients with anthracycline-induced atrial arrhythmias.

**Methods:**

This is a multicenter, randomized, double-blind, placebo-controlled clinical trial. A total of 152 eligible participants will be enrolled and randomly assigned to receive either WXKL or placebo for 3 months, followed by a 3-month follow-up period. The primary outcome is the proportion of patients free from atrial arrhythmias at 3 months, defined as the absence of atrial fibrillation (AF) episodes lasting ≥30 s or premature atrial contractions (PACs) ≥100 beats per 24 h on Holter monitoring. Secondary outcomes include AF burden, PACs count, echocardiographic parameters, cardiac biomarkers (cTnI and NT-proBNP), quality of life (FACT-G), functional status (ECOG), clinical symptom scores, and anxiety scores (SAS) at 3 and 6 months, as well as safety assessments.

**Discussion:**

This study is designed to provide high-level clinical evidence for the use of WXKL in the treatment of anthracycline-induced atrial arrhythmias. By incorporating both PACs and AF as a composite endpoint, the study aims to more comprehensively capture the overall burden and dynamic progression of atrial arrhythmias, and to inform cardio-protective strategies during cancer therapy.

**Trial registration:** http://itmctr.ccebtcm.org.cn/zh-CN, International Traditional Medicine Clinical Trial Registry Platform, ITMCTR2025000704.

## Introduction

Anthracycline has become cornerstone drug in the treatment of malignant tumors such as breast cancer, lymphoma, and leukemia due to their potent antitumor activity. Even amid the rapid advancement of immunotherapy and targeted therapies, their clinical role remains irreplaceable (1). However, the non-specific distribution characteristics of anthracycline result in significant cardiotoxicity alongside its antitumor effects, with this risk increasing as cumulative drug dose. Elderly patients, due to age-related decline in physiological function, exhibit a much higher incidence of cardiotoxicity compared to younger individuals (2–4). This anthracycline-induced cardiotoxicity (AIC) has long posed a significant challenge in clinical practice—not only severely limiting the clinical application of anthracycline but also profoundly impacting patient prognosis and quality of life. It represents a core challenge that must be overcome in the clinical use of anthracycline (5, 6). Clinically, AIC manifests in acute and chronic forms: Acute toxicity typically occurs within two weeks after a single dose or single course, primarily presenting as electrocardiographic (ECG) abnormalities and atrial or ventricular arrhythmias (7); chronic cardiotoxicity may develop months to years after treatment cessation, primarily manifesting as cardiomyopathy and heart failure (8). Research indicates that AIC is characterized by irreversible cardiac damage and myocardial cell death, with mechanisms involving: 1) oxidative stress and excessive ROS production; 2) topoisomerase II inhibition and DNA double-strand breaks leading to abnormal gene transcription and apoptosis; 3) activation of the apoptosis pathway due to mitochondrial dysfunction (6, 9). Arrhythmias, particularly atrial arrhythmias, are a major clinical manifestation of AIC that are intimately linked to the use of anthracycline. Data indicate that the overall risk of arrhythmias in patients receiving anthracycline-based chemotherapy increases to 90%, with the risk of atrial arrhythmias rising to 114% (3, 10). As a typical atrial arrhythmia, atrial fibrillation (AF) has a prevalence of up to 10.3% in this population, and the risk further increases with cumulative anthracycline dose (11). In addition to AF, premature atrial contractions (PACs) are also a common form of atrial arrhythmia in clinical practice. Previous studies have shown that frequent PACs not only reflect a state of atrial electrophysiological instability but also serve as an important precursor and predictive marker of AF, with their frequency positively correlated with the subsequent risk of AF (12, 13). Therefore, PACs and AF share a pathophysiological continuum and progressive relationship, jointly reflecting different stages of atrial electrical remodeling. For cancer patients, atrial arrhythmias exert multidimensional effects. They cause symptoms such as palpitations, chest tightness, and dizziness, diminishing quality of life. They also elevate the risk of stroke, heart failure, and mortality. Importantly, severe arrhythmias may necessitate interruption of chemotherapy, directly compromising anticancer efficacy (14). Consequently, Therefore, the effective identification and management of atrial arrhythmias induced by anthracycline drug are of great importance for ensuring the smooth implementation of anticancer therapy and improving patient prognosis (4, 15).

Nevertheless, current clinical management of anthracycline-induced atrial arrhythmias faces significant limitations. Conventional anti-arrhythmic drugs can provide some benefit, but they lack optimized regimens tailored to the unique physiological state of cancer patients—such as interactions with chemotherapeutic agents and multi-organ functional impacts. Additionally, certain drugs present issues like poor tolerability and pronounced side effects. Concurrently, traditional Chinese medicine (TCM) has gained increasing attention in the field of cardio-protection for cancer patients. Among these, Wenxin Keli (WXKL) holds particular promise. As the first Chinese patented anti-arrhythmic herbal formulation approved by the China Food and Drug Administration (16). WXKL is composed of five herbal ingredients: Codonopsis pilosula, Panax notoginseng, Polygonatum sibiricum, Nardostachys jatamansi, and Amber, which has the effects of replenishing qi, nourishing yin, and promoting blood circulation, and is widely employed in clinical practice for treating various arrhythmias (17). Mechanistic studies have confirmed that WXKL exerts its therapeutic effects through multiple pathways, including improving cardiomyocyte electrophysiology, counteracting oxidative stress, and regulating calcium homeostasis, demonstrating efficacy in AF and ventricular premature beats (18, 19). Furthermore, the agent exhibits a favorable safety profile and good tolerability with long-term use, underscoring its clinical utility (20). Although existing studies have preliminarily explored the the anti-arrhythmic mechanisms of WXKL, most are small-sample, single-center designs. In the field of cardio-oncology, no high-quality randomized controlled trials have yet validated its efficacy and safety for treating anthracycline-induced atrial arrhythmias, resulting in a lack of evidence-based support for its clinical application. Based on this, this study aims to conduct a multicenter, randomized, double-blind, placebo-controlled clinical trial to systematically evaluate the efficacy of WXKL in treating anthracycline-induced atrial arrhythmias. This study will not only fill the evidence-based gap in the field of onco-cardiology regarding the integrated traditional Chinese and Western medicine treatment of related complications, but also provide clinicians with a new cardio-protective strategy that combines efficacy and safety. It holds significant clinical importance for optimizing treatment regimens for patients undergoing anthracycline chemotherapy and enhancing the overall efficacy of comprehensive cancer therapy.

## Methods

### Study design and setting

This trial is a multicenter, double-blind, placebo-controlled study with two parallel groups designed to evaluate the efficacy of WXKL.The protocol was formulated in accordance with the SPIRIT statement and the Declaration of Helsinki (2013 Revision), as well as the Guidelines for Good Clinical Practice (GCP). The start and end of recruitment had been planned to be December 2025 and March 2028, respectively.

**Figure 1:**
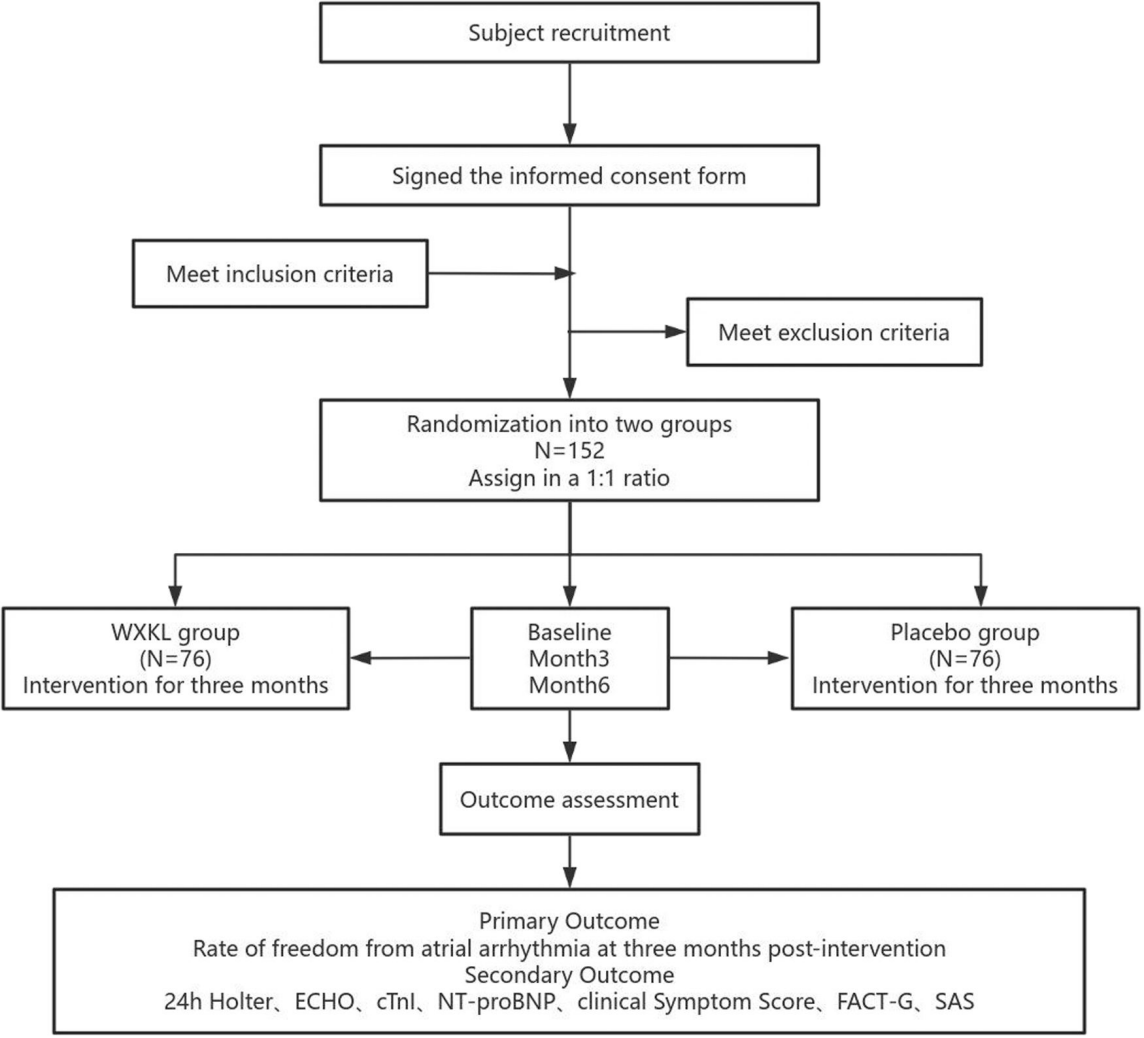
The participant flowchart. WXKL, Wenxin Keli; ECHO, echocardiography; cTnI, cardiac troponin I; NT-pro-BNP, N-terminal pro-B-type natriuretic peptide; FACT-G, functional assessment of cancer therapy for general. SAS, self-rating anxiety scale.

Before the experiment begins, researchers will conduct a training program to ensure that all participating medical staff fully understand each part of the trial. The trial will be conducted at multiple clinical centers in China, including Guang'anmen Hospital of the China Academy of Chinese Medical Sciences, Qilu Hospital of Shandong University, Xingtai People's Hospital, Affiliated Hospital of North China University of Science and Technology, Jinqiu Hospital of Liaoning Province, Tianjin Medical University Cancer Institute&Hospital, and Dezhou Second People's Hospital. After confirming eligibility and signing the informed consent form, 152 subjects receiving anthracycline treatment will be randomly allocated to either the WXKL or placebo groups in a 1:1 ratio. The study is divided into four phases: recruitment, allocation, intervention, and completion. Each participant will only be enrolled once. In addition to anthracycline chemotherapy and conventional anti-arrhythmic medications, participants will receive either WXKL or placebo treatment. During the study period, each participant will undergo three visits: baseline (Visit 1), month three (Visit 2), and month six (Visit 3). In Phase 1, participants are recruited from the oncology department, where they will undergo a physical examination and eligibility assessment. Those who match the inclusion criteria will receive a three-month pharmacological intervention. In Phase 2, following the completion of the intervention, participants will undergo clinical efficacy and safety assessments. Phase 3 is the follow-up period, during which participants will be followed up at the sixth month to gather their metrics again and assess clinical efficacy. The participant flowchart is shown in Figure 1, and the study schedule is shown in Table 1.

**Table 1:** Schedule of enrollment, intervention and assessment.

Study procedure		Intervention		
		Visit 1	Visit2	Visit3
Time point		baseline	month 3	month 6
Evaluation of inclusion/exclusion criteria		●		
Informed consent		●		
Basic information and demographics		●		
Past, personal and family history		●		
Cancer typing and staging		●		
Previous cancer treatment history		●		
Scale score	ECOG score	●	●	●
	Clinical symptom score	●	●	●
	FACT-G	●	●	●
	SAS	●	●	●
Complementary check-up	24 h Holter	●	●	●
	ECHO	●	●	●
	Complete blood count	●	●	●
	Liver function tests	●	●	●
	Renal function tests	●	●	●
	Electrolyte examination	●	●	●
	NT-pro-BNP	●	●	●
	cTnI	●	●	●
Merge Medication Records		●	●	●
Adverse Event and Significant Incident records			●	●
Test Reliability Evaluation		●		
Evaluation of Therapeutic Efficacy			●	●
Clinical Trial Summary Form				●
Summary Table (Test Report Attachment Area)		●	●	●

According to SPIRIT 2013 Statement: Defining Standard Protocol Items for Clinical Trials.

The study protocol has been approved by the Medical Ethics Committee of Guang'anmen Hospital, Chinese Academy of Medical Sciences, China (NO. 2024-194-KY-02) and registered on the International Traditional Medicine Clinical Trial Registry Platform (No. ITMCTR2025000704). All participants will provide written informed permission before any study-related operations are conducted. During the trial, any modifications to the study design will be promptly reported to the Ethics Committee.

### Participation and recruitment

#### Recruitment and consent

Potential participants will be initially identified via the hospital's electronic medical record system and routine outpatient visits. Clinical staff will approach individuals who meet the basic diagnostic criteria and invite them to join the study. Interested candidates will then undergo comprehensive screening, including detailed evaluations to confirm whether they satisfy the predefined inclusion and exclusion criteria. Once eligibility is confirmed, participants will receive complete study information and provide informed consent, thereby ensuring their voluntary involvement in the trial.

#### Inclusion criteria

The inclusion criteria for this study include the following conditions: 1) Age 18–80 years; 2) Having a confirmed pathological type, receiving chemotherapy for the first time, with an expected cumulative anthracycline dose ≥200 mg/m², and PAVs or AF after at least one cycle of anthracycline therapy, with ambulatory electrocardiography showing PAVs ≥100 beats per 24 h or meeting the diagnostic criteria for AF; 3) No history of PAVs or AF; 4) No prior history of chest radiotherapy, targeted therapy, or immunotherapy; 5) Eastern Cooperative Oncology Group (ECOG) performance status < 2; 6) Willing to be randomly assigned to either the WXKL group or the control group; 7) Voluntary participation in the trial and provision of signed informed consent.

#### Exclusion criteria

The exclusion criteria for this study include the following:1) Patients with inadequately controlled heart failure (left ventricular ejection fraction <50%); 2) Patients with QTc > 500 ms, a history of QTc prolongation (congenital or acquired), or severe bradyarrhythmia; 3) Patients who have experienced new-onset major vascular events within the past 3 months, such as myocardial infarction or cerebral infarction; 4) Patients with diagnosed cardiomyopathy (ischemic, dilated, restrictive, or hypertrophic) or moderate to severe valvular heart disease; 5) Patients with inadequately controlled blood pressure: systolic blood pressure ≥180 mmHg or diastolic blood pressure ≥ 110 mmHg, or systolic blood pressure <90 mmHg or diastolic blood pressure <60 mmHg; 6) Patients with chronic kidney disease and an estimated glomerular filtration rate (eGFR) <30 mL/min/1.73 m²; 7) Patients with serum potassium >5.5 mmol/L; 8) Patients with alanine aminotransferase (ALT) or aspartate aminotransferase (AST) levels exceeding three times the upper limit of normal; 9) Pregnant or lactating women; 10) Patients with allergies to or contraindications for the study drug components; 11) Patients currently participating in other clinical trials.

### Sample size calculation

To our knowledge, this is the first clinical trial to evaluate a TCM formulation for the treatment of anthracycline-induced atrial arrhythmias. Currently, no published data are specifically available for atrial arrhythmias induced by anthracycline. Therefore, the sample size estimation was based on existing studies of TCM in the treatment of AF or PVCs, together with clinical experience and expert consensus (21–23). We assumed that the proportion of patients free from atrial arrhythmias would be 29% in the WXKL group and 10% in the placebo group. Based on a two-sided significance level of 0.05 and a statistical power of 80%, the required sample size was calculated using PASS version 15. The calculation indicated that 68 participants were needed per group. Considering an anticipated dropout rate of 10% and rounding to whole numbers, the final sample size was set at 76 participants per group, resulting in a total of 152 participants to be enrolled in this trial.

### Randomization and blinding

This study will use centralized randomization. An independent statistical center will generate the randomization sequence. participants will be allocated in a 1:1 ratio to guarantee uniformity of baseline characteristics, and each participant will be assigned a trial code for identification. The randomization sequence details have been uploaded to the central randomization system. The trial is double-blinded for both investigators and participants. All trial medications—WXKL for the intervention group and placebo for the control group—are produced, packaged, and labeled by Shandong Buchang Pharmaceutical Co., Ltd. (Shandong, China). The placebo matches WXKL in appearance, texture, and weight. All packages are identical in outward form to maintain blinding throughout the study. In the event of a serious adverse event, the investigator may perform emergency unblinding for the affected participant and report accordingly to the ethics committee. After trial completion, statisticians will conduct the first unblinding and perform statistical analysis based on the grouped data. Following analysis, a second unblinding will reveal the final group allocations. All data collection and outcome assessments will be carried out by third-party personnel not involved in trial design or participant recruitment.

### Intervention measures

Participants will be randomly assigned to either the intervention group or the control group and will receive treatment for 3 months. During the study period, the use of any other traditional Chinese medicines with similar components to WXKL will be prohibited. From Day 1 after enrollment, participants in the intervention group will receive WXKL (National Drug Approval Number Z10950026), while those in the control group will receive a matched placebo. Both treatments will be administered orally as granules dissolved in warm water, one sachet (5 g) three times daily. Drug dispensing will be managed by designated personnel, with careful counting and documentation to ensure accurate allocation according to the randomization code. At the end of the trial, all unused medications will be collected and destroyed by designated staff.

Concomitant medications: (a) All participants will receive anthracycline-based chemotherapy according to standardized regimens prescribed by their treating physicians (24, 25). (b) According to current clinical guidelines, *β*-blockers are recommended as first-line agents for ventricular rate control in atrial arrhythmias. Therefore, for patients with symptoms or clinical indications, *β*-blockers will be administered as background antiarrhythmic therapy. The initiation, dose titration, and anticoagulation strategies will be determined based on patients' clinical status and relevant guidelines (26), to ensure both safety and consistency across study centers. (c) Concomitant use of medications for other comorbid conditions will be permitted. However, the use of other anti-arrhythmic drugs (except *β*-blockers) will be prohibited to minimize potential confounding effects. In addition, the use of cardio-protective agents specifically targeting anthracycline-induced cardiotoxicity, such as dexrazoxane, will not be allowed. All concomitant medications during the study will be recorded in detail in the case report forms (CRF).

Assigned interventions may be discontinued or modified in cases of serious adverse events, participant withdrawal, or clinical deterioration. Any such changes will be fully documented and submitted to the ethics committee for review.

### Measurements

The endpoint assessors are responsible for post-intervention follow-up. They conduct regular data collection and follow-up for all participants according to the study protocol schedule. Follow-up time points are at the end of the treatment period (3 months after enrollment) and 3 months after treatment completion (6 months after enrollment). The primary endpoint is evaluated based on 24 h Holter monitoring, assessing the absence of atrial arrhythmias in both groups at the end of treatment. In this study, the occurrence of atrial arrhythmias is defined as either a single episode of AF lasting ≥30 s, or ≥100 PAVs per 24 h on Holter monitoring (21). Holter data will be collected uniformly using a single-channel ambulatory electrocardiogram recorder (model: EPBB-PAH-1A; manufacturer: Shanghai Yuanxin Medical Technology Co., Ltd.; registration no.: 20212070427). At baseline and each follow-up visit, cardiac function will be assessed via echocardiography, as well as serum levels of cTnI and NT-proBNP. Patients' quality of life will be evaluated using the FACT-G scale, ECOG performance status, clinical symptom score, and the Self-Rating Anxiety Scale (SAS). Additionally, complications, adverse events, and laboratory parameters—including complete blood count, liver and kidney function, and electrolytes—will be recorded. All baseline data will be documented by the clinical investigators responsible for participant recruitment and intervention administration.

### Outcome evaluation

#### Primary outcome

The primary endpoint is the proportion of patients in both groups free of atrial arrhythmias 3 months after intervention, defined as the absence of any single episode of AF lasting ≥30 s or PAVs ≥100 beats per 24 h as detected by 24 h Holter monitoring.

#### Secondary outcome

Secondary outcomes include: (1) AF burden (defined as the cumulative duration of all AF episodes recorded on 24 h Holter monitoring) or the frequency of PAVs on Holter monitoring at 3 and 6 months after enrollment (21). (2) Echocardiographic parameters (LVEF, LVEDD, LVESD, LAD, RVD, E/A ratio) and levels of cTnI and NT-proBNP at 3 and 6 months. (3) Other secondary measures assessed at 3 and 6 months: the FACT-G questionnaire, ECOG performance status, clinical symptom scores, and the SAS scores.

#### Safety evaluation

Safety will be assessed through documentation of adverse events, physical examinations, vital signs, and laboratory tests, including complete blood count, electrolytes, liver function, and renal function. All laboratory results and reported adverse events will be used to evaluate the safety profile of WXKL. Any adverse event will be recorded promptly and in detail, noting the time of onset, duration, associated symptoms, relevant laboratory abnormalities, interventions taken, and outcome. These data will be reviewed to determine relatedness to WXKL. If a participant meets any predefined criteria for study discontinuation, the intervention will be stopped immediately, the treatment assignment will be unblinded as needed, and the participant will be managed to ensure a safe exit from the trial.

#### Baseline assessments

Baseline data of participants will be recorded before the initiation of treatment: (1) Demographic information: Gender, age, height, weight, medical history, personal history, family history, study center information, and medical record number. (2) Breast cancer or malignant lymphoma information: Pathological diagnosis, staging, chest radiation therapy (side and total dose), chemotherapy regimen, and cumulative dose of anthracycline drugs. (3) Comorbidities and related medications: Including hypertension, diabetes, dyslipidemia, coronary artery disease, and other medical history.

### Statistical analysis

All statistical analyses will be performed using SPSS (version 26.0, IBM) and R (version 4.1.1). All tests will be two-sided, and a *P* value <0.05 will be considered statistically significant. The primary analysis will follow the intention-to-treat (ITT) principle, including all randomized participants and analyzing them according to their assigned groups. A per-protocol (PP) analysis will be conducted as a sensitivity analysis, and the safety analysis will include all participants who received at least one intervention. Baseline characteristics will be described by group. Continuous variables will be presented as mean ± standard deviation or median (interquartile range), depending on their distribution, while categorical variables will be expressed as frequencies and percentages. All analyses will be reported in accordance with the CONSORT Statement.

The primary outcome is the proportion of patients free from atrial arrhythmias at 3 months after intervention, which is a binary variable. It will be analyzed using a generalized linear model (GLM) with a binomial distribution and a log link function. Treatment group and study center will be included as fixed effects, and pre-specified baseline covariates—including age, sex, cancer subtype, cumulative anthracycline dose, and medical history—will be further adjusted. Results will be presented as risk ratios (RR) with corresponding 95% confidence intervals. Secondary continuous outcomes, such as AF burden, premature PAVs counts, echocardiographic parameters, and relevant biomarkers, will be analyzed using linear models (LM), with adjustment for baseline values. For repeated measures data (e.g., baseline, 3 months, and 6 months), mixed-effects models will be applied to evaluate treatment effects over time.

The incidence of adverse events and serious adverse events will be summarized as frequencies and percentages, and compared between groups using the chi-square test or Fisher's exact test, as appropriate. Changes in laboratory parameters will be described descriptively and compared between groups when necessary. Pre-specified subgroup analyses will be conducted based on cumulative anthracycline dose (e.g., high vs. low dose). Interaction terms between treatment and subgroup will be included in the model, and the Wald test will be used to assess differences in treatment effects across subgroups. The normality of model residuals will be evaluated using Q–Q plots. If the normality assumption is violated, appropriate data transformations or robust statistical methods will be applied. Missing data will be handled using multiple imputation under the assumption of missing at random, and sensitivity analyses will be performed to assess the robustness of the results. No interim analysis is planned for this study.

### Data security monitoring and management

The study team provided coordination and daily support for the trial. The study leader, Yanwei Xing, supervised the study design and provided guidance throughout trial implementation. Study leader was responsible for all aspects of local organization, including identifying potential recruits and taking consensus. The trial steering committee is composed of the study leader (Xing), the site principal investigators ofrom each participating center, and co-investigators. Questions that arise during the research process will be submitted to the committee for decision-making. Finally, clinical research associates (CRAs) will supervise the study progress at any time and hold a meeting monthly. Prior to trial initiation, an independent Data Monitoring Committee (DMC) is convened, comprising five experts from the fields of oncology, cardiology, biostatistics, clinical trial methodology, and bioethics. This committee is responsible for safety monitoring, reviewing, and evaluating detailed information on adverse events across groups. Based on its assessments, the DMC may recommend early trial termination, suggest measures to mitigate risks, or advise adjustments to the study plan.

For every participant, whether completing the study or withdrawing, the CRF should be carefully completed in accordance with the study protocol after completing the Case Screening Form. The principal investigator at each site is responsible for the authenticity of the data collected at their center. Data management is uniformly conducted using the electronic data capture (EDC). Specifically, after a participant's visit, the investigator will enter data from the paper CRF into the EDC system. Following submission, the data will be reviewed by a monitor before being transferred to the data management company. If any queries arise regarding the CRF, the data manager will generate a Data Resolution Query (DRQ). This query will be sent to the investigator via the clinical monitor. The investigator is expected to respond promptly. The data manager will then update the database based on the investigator's response, issuing further DRQ if necessary. After blinded review and confirmation of the database's accuracy, the principal investigator and statistical analyst will lock the data. Once locked, the data files will no longer be modified, and the raw data will be retained for at least five years following the study's conclusion.

### Protocol amendments

Any protocol amendments will first be communicated to the funding agency, followed by notification to each study center. A copy of the revised protocol will be added to the Investigator Site File. All subsequent major protocol amendments will be documented and submitted to the Ethics Committee for review prior to implementation. The principal investigator at each center is responsible for ensuring all subsequent amendments receive the necessary approvals.

### Dissemination plans

The results of this study will be widely disseminated through a series of peer-reviewed publications; presentations at local, national, and international academic conferences and reports to funding agent. In addition, a summary of the primary outcome findings will be created in English and Chinese and shared with the study participants.

## Discussion

Anthracycline-induced cardiotoxicity remains a challenging clinical issue in cancer treatment. Despite the unshakable role of these agents in treating breast cancer, lymphoma, and other malignancies, their associated cardiac injury—particularly early-onset atrial arrhythmias—significantly impacts treatment tolerance and long-term patient outcomes (27). Effective management of this complication is not only a vital mission for cardio-oncology but also an essential requirement for achieving comprehensive, high-quality survival for cancer patients (28). Previously, clinical management strategies for such arrhythmias primarily relied on conventional anti-arrhythmic drugs, but their application in the specialized population of cancer patients often presents challenges. Moreover, patients frequently exhibit reduced tolerance to standard pharmacotherapy due to metabolic disturbances from the cancer or its treatment, increased multi-organ burden, and complex drug interactions. Furthermore, the potential negative inotropic effects or arrhythmogenic risks associated with certain drugs raise concerns about the safety of their long-term use.

As an important alternative and complementary therapy, WXKL demonstrate significant potential and clinical value in preventing and treating such complications due to their multi-component, multi-target, and holistic regulatory properties (29, 30). Extensive prior research indicates that this medication exerts its anti-arrhythmic effects through multiple mechanisms, including anti-oxidant stress, energy metabolism regulation, anti-inflammatory and anti-apoptotic actions, and modulation of sodium-calcium channels (31–34). This provides theoretical support for its application in treating anthracycline-induced atrial arrhythmias. However, in clinical practice to date, no randomized controlled trials have specifically investigated the efficacy and safety of WXKL in anthracycline-induced atrial arrhythmias. To address this evidence gap, the research team designed this multicenter, randomized, double-blind, placebo-controlled trial. It aims to overcome limitations of previous studies, generate high-level evidence for WXKL in cardio-oncology, specifically for anthracycline-induced atrial arrhythmias, and to further validate its efficacy and safety.

The trial employs a multidimensional assessment strategy. Efficacy and subclinical cardio-protection will be objectively evaluated using 24 h Holter monitoring, echocardiography, and circulating biomarkers (NT-proBNP, cTnI). Concurrently, patient-reported outcomes—including the FACT-G questionnaire, ECOG performance status, clinical symptom scores, and SAS scores—will systematically capture improvements in quality of life, providing a congruent and comprehensive view of cardiac benefit. The multicenter design enhances the generalizability of the findings and minimizes confounding from local practice patterns or individual patient characteristics. If the study yields positive results, it will not only provide a high-quality, evidence-based cardio-protective regimen for cancer chemotherapy patients to delay the onset of malignant cardiac events, but also help advance the shift in cancer treatment from a “disease-centere” to a “patient-centered” model. By proactively managing treatment-related toxicities, this approach would tangibly improve the long-term quality of life and overall prognosis for cancer patients, contributing significant evidence-based support for TCM to clinical practice in cardio-oncology.

This study also has several limitations. First, arrhythmias assessment is based on 24 h Holter monitoring. Although this method is widely used in clinical research, its relatively short monitoring duration may lead to underdetection of intermittent atrial arrhythmias; extending the monitoring period or using implantable devices may improve detection rates (35). However, in multicenter randomized controlled trials, 24 h Holter monitoring remains a practical approach due to its feasibility, high patient compliance, non-invasiveness, and relatively low cost. Moreover, it can provide standardized and reproducible quantitative metrics, such as AF episodes and PAVs burden, and is therefore still commonly used in clinical trials (36). Second, although most clinical trials currently define AF as a single episode lasting ≥30 s (37), there is no universally accepted definition for frequent PAVs (38, 39). Previous studies have used varying thresholds for PAVs; for example, some have defined it as ≥500 beats per 24 h, whereas others have used ≥ 720 beats per 24 h or ≥1,000 beats per 24 h (22, 38). Although no consensus cutoff has been established, the clinical significance of frequent PAVs is primarily reflected in the strong association between increasing PAVs burden and the risk of AF (12). Based on these considerations, in the present study design, we integrated evidence from the literature and expert opinions in the field of cardio-oncology, while taking into account the overall condition and treatment tolerance of patients undergoing chemotherapy. A relatively conservative threshold ((≥100 beats per 24 h) was adopted as the inclusion criterion. This approach may enhance the sensitivity for detecting potential anthracycline-induced atrial arrhythmias within a limited monitoring period, thereby enabling earlier identification of clinically meaningful atrial electrical abnormalities. Nevertheless, it should be acknowledged that the choice of different thresholds may influence event detection rates and the comparability of study results, which represents an inherent limitation. Further prospective studies are warranted to establish the optimal clinical cutoff for frequent PAVs in the field of cardio-oncology. Finally, due to the lack of published data specifically targeting this study population, the assumptions used for sample size estimation were derived from studies involving AF and related PAVs, combined with clinical experience and expert opinion, which may introduce uncertainty. Larger-scale trials are therefore needed to further validate the clinical benefits of WXKL.

## Ethics statement

The studies involving humans were approved by Medical Ethics Committee, Guang'anmen Hospital, China Academy of Traditional Chinese Medicine. The studies were conducted in accordance with the local legislation and institutional requirements. The participants provided their written informed consent to participate in this study.
